# Correction: California condor microbiomes: Bacterial variety and functional properties in captive-bred individuals

**DOI:** 10.1371/journal.pone.0230738

**Published:** 2020-03-16

**Authors:** Lindsey Jacobs, Benjamin H. McMahon, Joel Berendzen, Jonathan Longmire, Cheryl Gleasner, Nicolas W. Hengartner, Momchilo Vuyisich, Judith D. Cohn, Marti Jenkins, Andrew W. Bartlow, Jeanne M. Fair

The eighth author’s middle initial is incorrect. The correct name is: Judith D. Cohn.
