# Supplementary figures and images for: California condor microbiomes: Bacterial variety and functional properties in captive-bred individuals
Source: PLoS One. 2019 Dec 11;14(12):e0225858. doi: 10.1371/journal.pone.0225858 (PMC6905524; doi:10.1371/journal.pone.0225858)

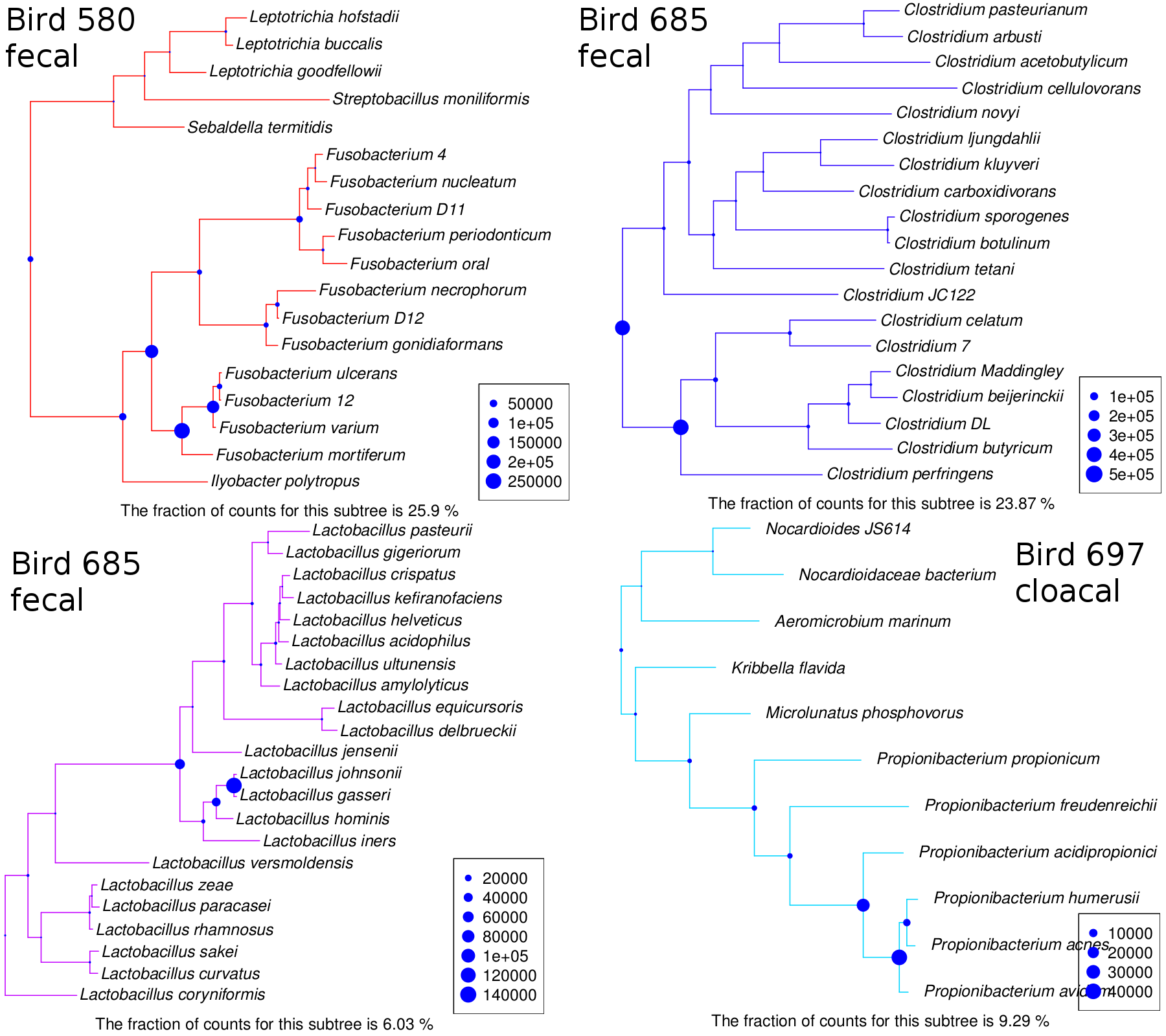

Supplement: S1 Fig — Close-ups on reference tree of reads recruited for four representative samples, highlighting a region of the phylogenetic tree near (top-left) Fusobacterium mortiferum, (top-right) Clostridium perfringens, (bottom-left) Lactobacillus johnsonii and (bottom-right) Propionibacterium avidum. Selected reads from a phylogenetic marker gene (RNA Polymerase) were examined with Blastn or Blastp against the non-redundant databases at NCBI to identify the most closely related reference organism. (PNG) [file pone.0225858.s001.png]

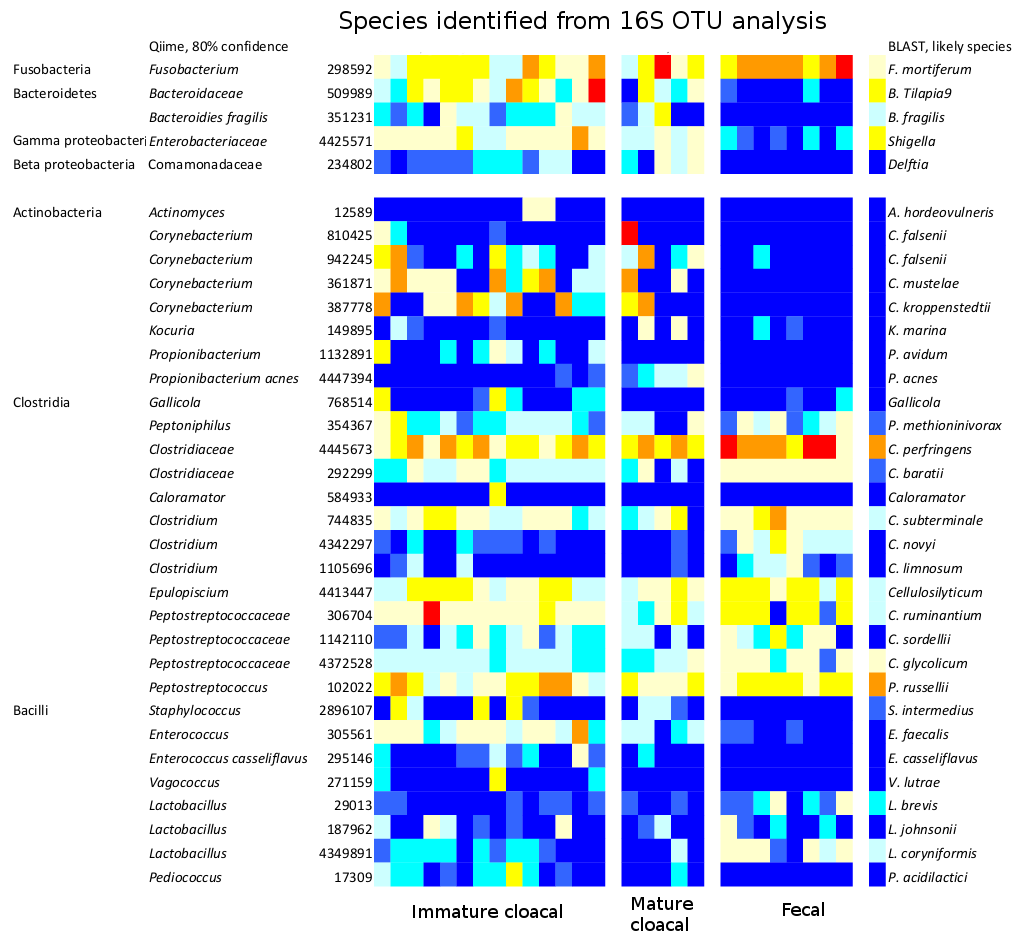

Supplement: S2 Fig — The fraction of the sample represented in the indicated OTU is shown as a heat map with a factor two ratio between adjacent colors, which run from dark blue to red. The rows are sorted phylogenetically, while the columns are in the same order as in Table 1 and Fig 1, but with the two samples for which the 16S samples failed left out of the heat map. The columns represent different samples in the same order as in Fig 2, and the rows represent the different nodes of the phylogeny. To the left of the heat map, we provide the most specific phylogenetic assignment QIIME made at the 80% confidence level, grouped phylogenetically in the same order as in Fig 2. These phylogenetic assignments correspond to the 16S sequences deposited in the Sequence Read Archive for this study. Representative 16S sequences are provided in S2 File, and are numbered according to the identifiers provided on the left of the heat map above. To the right of the heat map are the species names inferred from the 16S sequence and knowledge of what species were found with the RNA polymerase nucleotide reads identified by Sequedex from the shotgun sequencing data Similar to the process used to make Fig 2, representative sequences for each OTU were compared to the Ribosomal DataBase project using BLASTN to identify the species provided to the right of the Figure. (PNG) [file pone.0225858.s002.png]

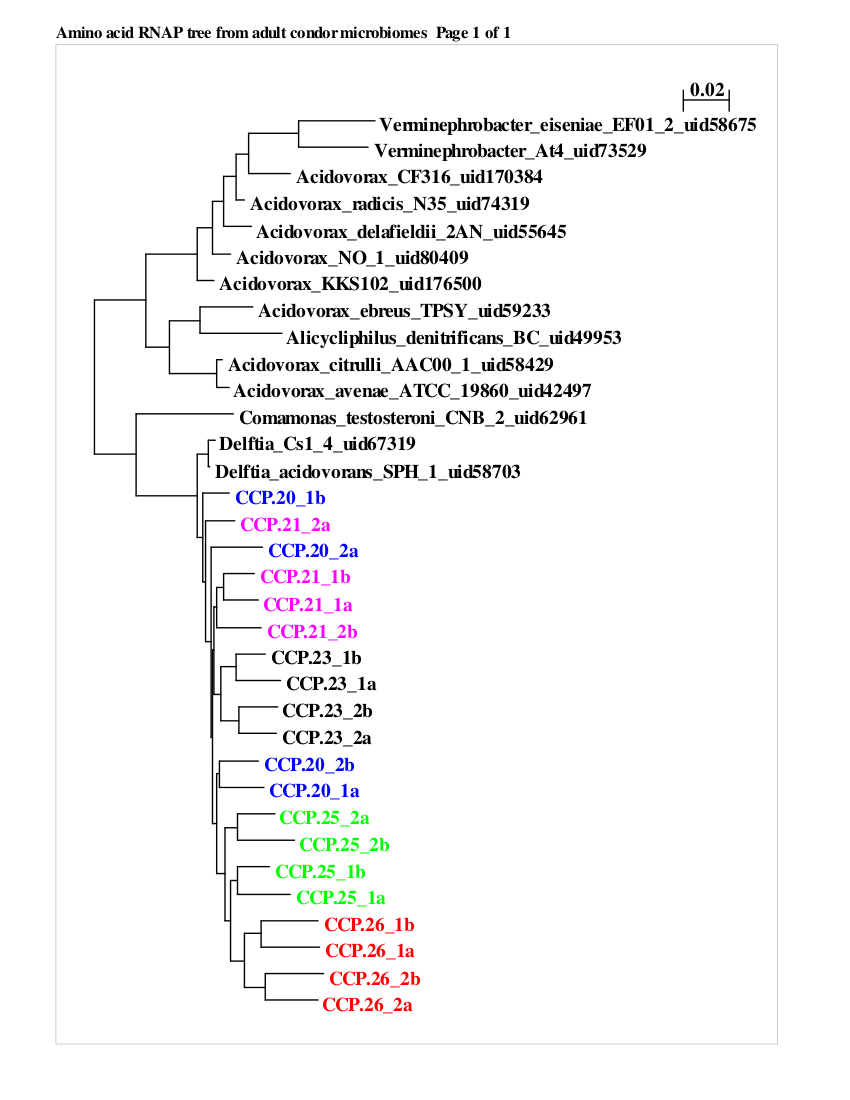

Supplement: S3 Fig — Phylogenetic tree of the RNA polymerase genes from reference genomes phylogenetically near to Delftia, and four assembled RNA polymerase genes from each of five mature cloacal samples. The sample numbers ‘CCP.xx’ refer to Table 1, while the ‘1’ or ‘2’ refer to forward and reverse reads, which were kept separate. Two equivalent assemblies were made to both the forward and reverse reads, and they are referred to as ‘a’ and ‘b’. The alignment used to compute the tree is provided as a Supplementary text file. S3 Fig shows a phylogenetic tree resulting from such analysis. (PNG) [file pone.0225858.s003.png]

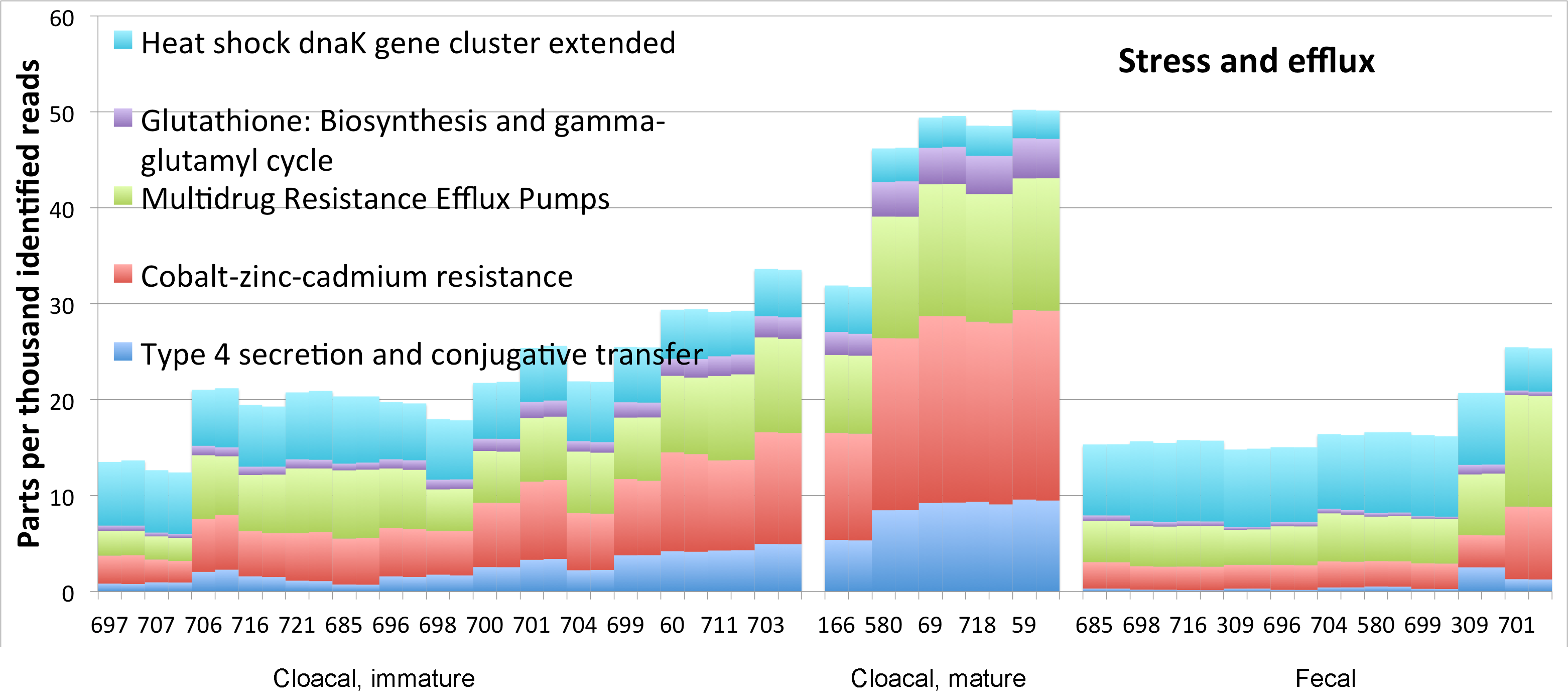

Supplement: S4 Fig — Selected subsystems distinctive of the stress response and efflux of toxic compounds are plotted, with the y-axis measuring the abundance of functionally identified reads associated with the particular subsystem, for each of the samples. (TIF) [file pone.0225858.s004.tif]

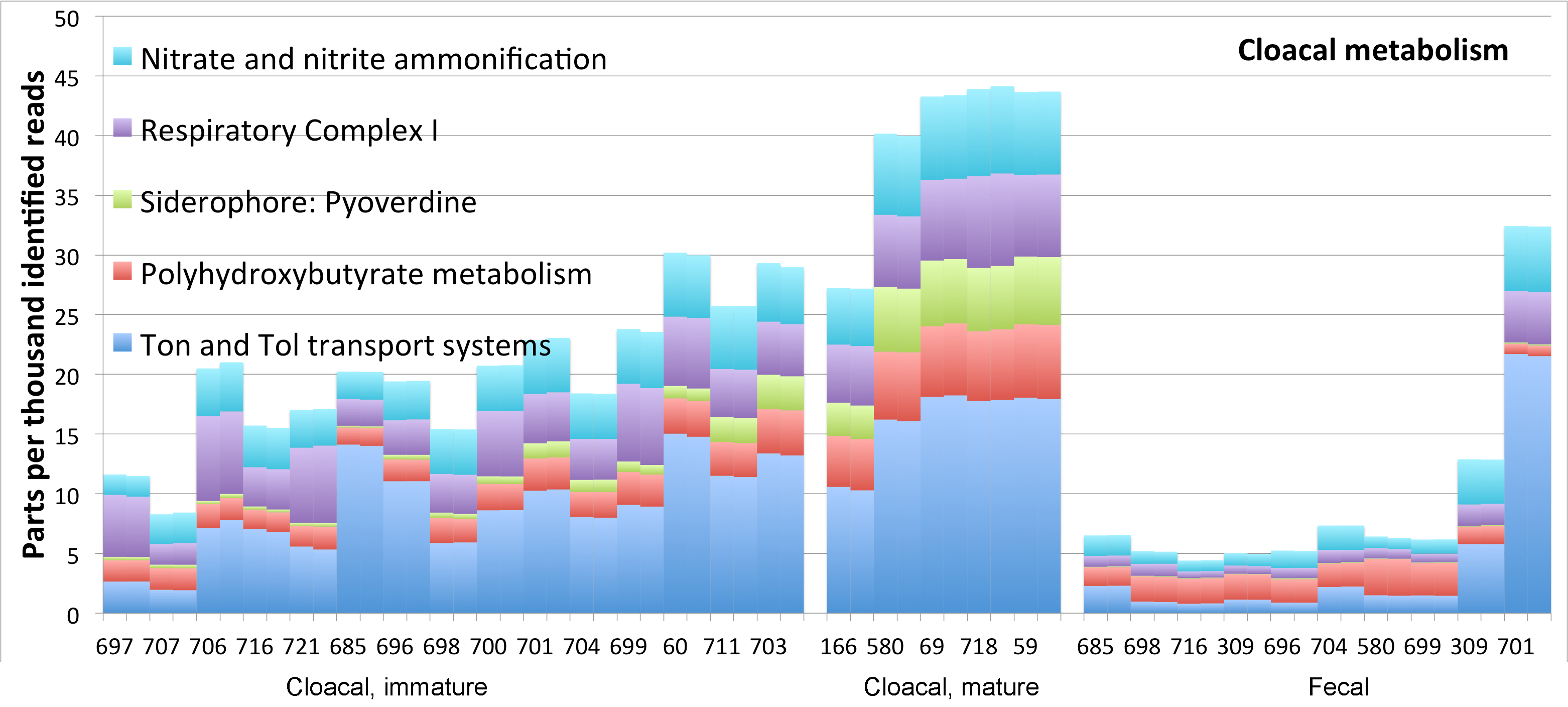

Supplement: S5 Fig — Selected subsystems distinctive of the fecal microbiome are plotted, with the y-axis measuring the abundance of functionally identified reads associated with the particular subsystem, for each of the samples. (TIF) [file pone.0225858.s005.tif]
