# Supplementary material for: California condor microbiomes: Bacterial variety and functional properties in captive-bred individuals
Source: PLoS One. 2019 Dec 11;14(12):e0225858. doi: 10.1371/journal.pone.0225858 (PMC6905524; doi:10.1371/journal.pone.0225858)
Supplement: S1 Text — (DOCX) [file pone.0225858.s009.docx]

**Supporting Information**

**Function Genes**

Acetyl-CoA fermentation to butyrate, in the Fermentation section of the Carbohydrates rollup category, involves a variety of transferases and dehydrogenases [1]. Butyrate can moderate immune response by acting as an inhibitor of pro-inflammatory pathways [2, 3], can restore tight junction barriers [4], can modulate epigenetics [3], and can act as an energy source. Genes involved with butyrate production are linked to *C. perfringens*, a bacterium we found abundant in condor fecal samples [5]. The presence of this subsystem indicates butyrate is present, can provide energy for the growth of microbiota, and likely correlates with good health of the condors.

Methionine degradation, in the Amino acids and derivatives rollup category, involves methionine transporters and a variety of lyases and synthetases [6]. Past studies have presented methionine as toxic in excessive quantities [7]. A more recent study agrees but specifies that methionine is not toxic except at levels more than 5 times normal intake in adult humans [8]. Degradation of methionine keeps this amino acid below dangerous levels and allows for production of cysteine, another amino acid [8, 9]. This produced cysteine is necessary for the synthesis of glutathione, a molecule important for antioxidant defense and cell signaling [9, 10]. Methionine degradation pathways have been associated with healthy humans, indicating condors with this subsystem are healthy [11].

Glycolysis and gluconeogenesis in the central carbohydrate section of the Carbohydrates rollup category contains genes such as phosphofructokinase and pyruvate kinase [12]. Gluconeogenesis produces glucose, which is then used in glycolysis to produce two molecules of ATP (energy) from every glucose molecule. Gluconeogenesis dominates during starvation while glycolysis is abundant in the fed state, so that energy is available in either condition. Although such core metabolic genes are present across the bacterial kingdom, their relative enrichment in the fecal samples likely indicates the predominance of rapidly growing organisms with relatively small genomes, as expected in the nutrient-rich, temperature-controlled, anaerobic *environment* of the avian gut.

Translation elongation factors: bacterial, Subsystem si_0668, in the Protein biosynthesis section of the Protein metabolism rollup category contains elongation factors Tu, Ts, G, and P. Factor Tu is a G-protein that binds aminoacyl-tRNA to the A-site of the ribosome, factor G moves the peptidyl-tRNA from the A-site to the P-site of the ribosome, and factor Ts regenerates an active factor Tu from its inactive form. Factor P prevents ribosomal stalling at polyproline stretches, thus enabling the translation of polyproline-containing proteins [13]. These bacterial translation elongation factors are important in protein synthesis [14], and like the glycolysis genes described above, are indicative of rapidly growing bacteria in the nutrient rich environment of the avian gut.

Subsystem si_0085, Maltose and maltodextrin utilization, in the Di- and oligosaccharides section of the Carbohydrates rollup category contains a maltodextrin-specific transporter and related enzymes [15]. Maltose and maltodextrin can be the primary carbon and energy sources utilized by bacteria such as *Bacteroidetes*. Additionally, the gram-positive bacterial genus *Listeria* contains gene products with high similarity to the maltodextrin transporter and utilization system of *B. subtilis* [16]*.* Thus, we expect that maltose and maltodextrin are important nutrients for the condor gut microbiome. Statistically important aspects of cloacal metabolism are of two types, those associated with metabolism, and those associated with response to stress. We start with the metabolic subsystems shown in Figure 6.

Subsystem si_593, Nitrate and nitrite ammonification, in the Nitrogen metabolism category contains nitrate and nitrite reductases as well as nitrogen transporter proteins [17]. Nitrogen is an essential element of life, but different types of bacteria obtain their nitrogen by different mechanisms [18]. The nitrate and nitrite ammonification subsystem produces ammonium by reducing nitrate to nitrite and nitrite to ammonium [18]. The importance of nitrate and nitrite ammonification to wastewater biofilm bacteria, *Delftia* and *Comamonas*, is described above in the mucosal microbes subsection of our discussion on the phylogenetic composition of our samples.

Subsystem si_0812, Respiratory complex I, in the Electron donating reactions section of the *Respiration* rollup category contains NADH dehydrogenase and ubiquinone [19]. This complex is a proton pump which generates a proton motive force across the bacterial membrane and through this synthesizes ATP [20]. Three protons and two electrons are transferred in each cycle of the proton pump, producing one ATP molecule for every two electrons. Respiratory complex I is central to aerobic metabolism, and is relatively enriched in the cloacal samples because of the anaerobic environment of the fecal samples.

Pyoverdine, subsystem si_0473, in the Siderophores section of the Iron acquisition rollup category, contains peptide synthases, siderophore receptors, and transporters [21]. Siderophores such as pyoverdine have a high affinity for iron and enable bacteria to obtain this element [22]. Most bacteria need iron for respiration and metabolic processes, and avoid the toxicity of excess amounts through iron storage or export. Binding excess iron is also beneficial because freely available iron can stimulate virulence in pathogenic bacterial species.

Polyhydroxybutyrate metabolism, subsystem si_0463, in the Fatty acids, lipids, and isoprenoids rollup category, contains microorganisms that synthesize the energy storage molecule polyhydroxybutyrate (PHB) [23]. This molecule is made from acetyl-CoA using three enzymatic steps. PHB is produced by various microbial species if certain nutrient conditions are met or if stressful conditions are present. The energy from this PHB energy molecule can be used if common energy sources are lacking.

Ton and tol transport systems, subsystem si_0526*,* in the Membrane transport rollup category contains ton and tol associated proteins [24]. These proteins are involved with bacterial iron acquisition and use [25]. They are also involved with cell invasion: colicins are bacterial toxins that use ton and tol transport systems to move through target cell envelopes before infecting and killing the cells [24]. Their presence in the cloacal samples may indicate the predominance of specialist bacteria that can utilize more complex nutrients, in comparison to the fecal bacteria. Functional categories related to stress and efflux that are statistically enriched in the cloaca are shown in Figure 7.

DnaK heat shock cluster, subsystem si_0851, in the Stress response rollup category contains dnaJ (heat-shock protein 40), dnaK (heat-shock protein 70), and GrpE [17]. DnaK proteins may be active constantly or be expressed in response to various stresses [26]. DnaJ proteins stimulate the ATP hydrolysis that is essential for the activity of dnaK, through this regulating protein translation, folding, unfolding, translocation, and degradation. These stress response genes may indicate the presence of phages, toxins, antimicrobial compounds, or host immune activity in the mucosal environment of the cloaca.

Subsystem si_0859, Glutathione: biosynthesis and gamma-glutamyl cycle, in the Oxidative stress section of the Stress response rollup category contains glutathione synthase and gamma-glutamyl transpeptidase [27]. Gamma-glutamyl transpeptidase is an enzyme of the gamma-glutamyl cycle that performs various functions, including breaking down extracellular glutathione and providing the rate-limiting substrate cysteine for intracellular synthesis of glutathione. Synthesis of glutathione is done by the gamma-glutamyl cycle through two ATP-dependent reactions, the second of which is catalyzed by glutathione synthase. Glutathione itself is important for many biological functions including cell signaling and detoxification. In situations of severe oxidative stress, maintenance of the glutathione poll is important for the health of cells. Like the heat shock cluster, this indicates that the cloacal environment includes oxidative stress.

Subsystem si_0939, Multidrug resistance efflux pumps in the Resistance to antibiotic compounds and toxins section of the Virulence, disease, and defense rollup category contains efflux-pump proteins from five families, including the ATP-blinding cassette (ABC) family and the multidrug and toxic-compound extrusion (MATE) family [28]. These efflux proteins grant resistance by moving drugs, toxins, and host molecules out of bacterial cells. Different pumps are specific for one or multiple compounds, so it is not clear at this level the specific nature of the ions or small molecules providing stress in the cloacal environment.

Subsystem si_0929, Cobalt-zinc-cadmium resistance in the Resistance to antibiotic compounds and toxins section of the Virulence, disease, and defense rollup category involves metal resistance mechanisms such as efflux pumps and enzymatic detoxification [29]. When metal ions are excessively abundant in bacterial cells, specific efflux pumps can be synthesized to export nonessential metals. Bacterial metal resistance may also be increased if the bacterial cell forms a resistance operon to detoxify a metal when that metal’s presence is detected. This subsystem indicates metal is present in condors.

Subsystem si_0906, Type 4 secretion and conjugative transfer in the Virulence, disease, and defense rollup category contains the type IV secretion system [30]. The type IV system exports proteins, toxins, and DNA conjugation intermediates. Some of the proteins secreted into host cells are involved in virulence, while others are exported with DNA and help process this DNA to form a conjugation intermediate. The type IV system may also export molecules that contribute to the biofilm surrounding the organism.

**Supporting Information Captions**

**Fig. S1.** Phylogenetic Trees of Four Bacterial Genera in Condors. close-ups on reference tree of reads recruited for four representative samples, highlighting a region of the phylogenetic tree near (top-left) *Fusobacterium mortiferum,* (top-right) *Clostridium perfringens,* (bottom-left) *Lactobacillus johnsonii* and (bottom-right) *Propionibacterium avidum*. Selected reads from a phylogenetic marker gene (RNA Polymerase) were examined with Blastn or Blastp against the non-redundant databases at NCBI to identify the most closely related reference organism.

**Fig. S2.** Results of 16S OTU analysis. The fraction of the sample represented in the indicated OTU is shown as a heat map with a factor two ratio between adjacent colors, which run from dark blue to red. The rows are sorted phylogenetically, while the columns are in the same order as in Table 1 and Figure 1, but with the two samples for which the 16S samples failed left out of the heat map. The leftmost label is the phylum classification, then the QIIME-assigned taxonomic label and taxon ID, corresponding to the 16S sequences deposited in the Sequence Read Archive for this study. To the right of the heat map are the species names inferred from the 16S sequence and knowledge of what species were found with the RNA polymerase nucleotide reads identified by Sequedex from the shotgun sequencing data.

**Fig. S3.** 16S Rarefaction Curves. Within-sample diversity, estimated by OTU analysis of 16S sequences with QIIME [31]. The rarefaction curves are computed by sampling the number of 16S sequences indicated on the x-axis and counting the number of distinct observed species, plotted on the y-axis. There is a factor four dispersion of the number of species found in the immature cloacal samples (the average is plotted).

**Fig. S4.** Phylogeny of Condors’ Microbiomes. Phylogenetic tree of the RNA polymerase genes from reference genomes phylogenetically near to Delftia, and four assembled RNA polymerase genes from each of five mature cloacal samples. The sample numbers ‘CCP.xx’ refer to Table 1, while the ‘1’ or ‘2’ refer to forward and reverse reads, which were kept separate. Two equivalent assemblies were made to both the forward and reverse reads, and they are referred to as ‘a’ and ‘b’. The alignment used to compute the tree is provided as a Supplementary text file.

**Fig. S5.** Principal Component Analysis Using Sequedex. Principal value decomposition of samples on the basis of profile across 963 SEED functional categories was performed in order to identify significant changes between sample-types. Three groups of samples emerged from this analysis, which we label according to the dominant type of sample: Fecal and mature and immature cloacal samples, with the transcriptome and several others appearing between clusters, perhaps indicating a mixture of sample-types.. These categories are used in the analysis presented in the two plots to the right. Placement of microbiomes on first two principal components when decomposed by normalized functional profiles determined with Sequedex using the SEED classification of protein function, as described in text. Three groups are defined for further study. Data sets indicating fecal samples are colored red, while cloacal samples are colored by age. Labels indicate condor number, with lower numbers indicating earlier births.

**S1 File.** Spreadsheet with six tabs (a-f) of phylogenetic and functional classifications by various metrics. **(a)** Phylogenetic rollup using Sequedex to classify shotgun metagenomic reads. **(b)** Normalized counts of reads assigned to each node of the Sequedex phylogenetic tree. **(c)** Operational taxonomic unit (OTU) counts for the 16S reads from each sample. (d) Functional rollup using Sequedes to classify shotgun metagenomic reads according to the high-level SEED classifications. **(e)** Normalized counts of shotgun metagenomic reads assigned to each SEED subsystem. **(f)** Normalized counts of shotgun metagenomic reads assigned to each Pfam family.

**S2 File.** Representative 16S sequences of OTUs, identified by the labels provided in Supplementary information SI1c, provided in fasta format.

**S3 File.** Representative genes assembled from open reading frames identified by Sequedex, in a spreadsheet format.

**Supplemental References**

1. Caspi R, Billington R, Ferrer L, Foerster H, Fulcher CA, Keseler IM, et al. The MetaCyc database of metabolic pathways and enzymes and the BioCyc collection of pathway/genome databases. Nucleic Acids Res. 2016;44:D471-80.

2. Zhang WH, Jiang Y, Zhu QF, Gao F, Dai SF, Chen J, et al. Sodium butyrate maintains growth performance by regulating the immune response in broiler chickens. Br Poult Sci. 2011;52:292-301.

3. Moquet PCA, Onrust L, Van Immerseel F, Ducatelle R, Hendriks WH, Kwakkel RP. Importance of release location on the mode of action of butyrate derivatives in the avian gastrointestinal tract. 2016;72:61-80.

4. Ploger S, Stumpff F, Penner GB, Schulzke JD, Gabel G, Martens H, et al. Microbial butyrate and its role for barrier function in the gastrointestinal tract. Ann Ny Acad Sci. 2012;1258:52-9.

5. Vital M, Gao JR, Rizzo M, Harrison T, Tiedje JM. Diet is a major factor governing the fecal butyrate-producing community structure across Mammalia, Aves and Reptilia. Isme J. 2015;9:832-43.

6. Livesey G. Methionine Degradation - Anabolic and Catabolic. Trends Biochem Sci. 1984;9:27-9.

7. Acar N, Barbato GF, Patterson PH. 2. the effect of feeding excess methionine on live performance, carcass traits, and ascitic mortality. Poult Sci. 2001;80:1585-9.

8. Garlick P. Toxicity of Methionine in Humans1. The Journal of Nutrition. 2006;136:1722S-5S.

9. Stipanuk M, Ueki I. Dealing with methionine/homocysteine sulfur: cysteine metabolism to taurine and inorganic sulfur. Journal of Inherited Metabolic Disease. 2011;34:17-32.

10. Wilber A. Glutathione Dietary Sources, Role in Cellular Functions and Therapeutic Effects. Ebooks C, editor. Hauppauge: Hauppauge : Nova Science Publishers, Inc.; 2014.

11. Hollister EB, Gao C, Versalovic J. Compositional and Functional Features of the Gastrointestinal Microbiome and Their Effects on Human Health. Gastroenterology. 2014;146:1449-58.

12. Berg JM TJ, Stryer L. Biochemistry. 5th ed. New York: W.H. Freeman; 2002.

13. Ude S, Lassak J, Starosta AL, Kraxenberger T, Wilson DN, Jung K. Translation Elongation Factor EF-P Alleviates Ribosome Stalling at Polyproline Stretches. Science. 2013;339:82-5.

14. Mitchell A, Chang HY, Daugherty L, Fraser M, Hunter S, Lopez R, et al. The InterPro protein families database: the classification resource after 15 years. Nucleic Acids Res. 2015;43:D213-21.

15. Schonert S, Seitz S, Krafft H, Feuerbaum EA, Andernach I, Witz G, et al. Maltose and maltodextrin utilization by Bacillus subtilis. Journal of Bacteriology. 2006;188:3911-22.

16. Gopal S, Berg D, Hagen N, Schriefer EM, Stoll R, Goebel W, et al. Maltose and Maltodextrin Utilization by Listeria monocytogenes Depend on an Inducible ABC Transporter which Is Repressed by Glucose. Plos One. 2010;5.

17. Overbeek R, Begley T, Butler RM, Choudhuri JV, Chuang HY, Cohoon M, et al. The subsystems approach to genome annotation and its use in the project to annotate 1000 genomes. Nucleic Acids Research. 2005;33:5691-702.

18. Simon J, Klotz MG. Diversity and evolution of bioenergetic systems involved in microbial nitrogen compound transformations. Biochimica et Biophysica Acta (BBA) - Bioenergetics. 2013;1827:114-35.

19. Richardson DJ. Bacterial respiration: a flexible process for a changing environment. Microbiol-Uk. 2000;146:551-71.

20. Wikström M, Hummer G. Stoichiometry of proton translocation by respiratory complex I and its mechanistic implications. P Natl Acad Sci USA. 2012;109:4431-6.

21. Schalk IJ, Guillon L. Pyoverdine biosynthesis and secretion in Pseudomonas aeruginosa: implications for metal homeostasis. Environ Microbiol. 2013;15:1661-73.

22. Kortman GAM, Raffatellu M, Swinkels DW, Tjalsma H. Nutritional iron turned inside out: intestinal stress from a gut microbial perspective. Fems Microbiol Rev. 2014;38:1202-34.

23. Singh P, Parmar N. Isolation and characterization of two novel polyhydroxybutyrate (PHB)-producing bacteria. African journal of biotechnology. 2013;10:4907-19.

24. Lloubes R, Goemaere E, Zhang X, Cascales E, Duche D. Energetics of colicin import revealed by genetic cross-complementation between the Tol and Ton systems. Biochem Soc T. 2012;40:1480-5.

25. Danese I, Haine V, Delrue RM, Tibor A, Lestrate P, Stevaux O, et al. The Ton system, an ABC transporter, and a universally conserved GTPase are involved in iron utilization by Brucella melitensis 16M. Infect Immun. 2004;72:5783-90.

26. Qiu XB, Shao YM, Miao S, Wang L. The diversity of the DnaJ/Hsp40 family, the crucial partners for Hsp70 chaperones. Cell Mol Life Sci. 2006;63:2560-70.

27. Zhang HQ, Forman HJ, Choi J. gamma-Glutamyl transpeptidase in glutathione biosynthesis. Method Enzymol. 2005;401:468-83.

28. Piddock LJV. Multidrug-resistance efflux pumps ? not just for resistance. Nat Rev Micro. 2006;4:629-36.

29. Bruins MR, Kapil S, Oehme FW. Microbial Resistance to Metals in the Environment. Ecotoxicology and Environmental Safety. 2000;45:198-207.

30. Christie PJ, Vogel JP. Bacterial type IV secretion: conjugation systems adapted to deliver effector molecules to host cells. Trends Microbiol. 2000;8:354-60.

31. Caporaso JG, Kuczynski J, Stombaugh J, Bittinger K, Bushman FD, Costello EK, et al. QIIME allows analysis of high-throughput community sequencing data. Nat Methods. 2010;7:335-6.
